# Circular RNA hsa_circ_0003823 promotes the Tumor Progression, Metastasis and Apatinib Resistance of Esophageal Squamous Cell Carcinoma by miR-607/CRISP3 Axis

**DOI:** 10.7150/ijbs.76096

**Published:** 2022-09-21

**Authors:** Yu-Ming Wang, Qi-Wu Zhao, Zhi-Yong Sun, Hai-Ping Lin, Xin Xu, Min Cao, Yu-Jie Fu, Xiao-Jing Zhao, Xiu-Mei Ma, Qing Ye

**Affiliations:** 1 Department of Thoracic Surgery, Renji Hospital, School of Medicine, Shanghai Jiao Tong University, Shanghai 200127, P.R. China.; 2 Department of General Surgery, Ruijin Hospital, School of Medicine, Shanghai Jiao Tong University, Shanghai 200000, P.R. China.; 3 Department of Radiation Oncology, Renji Hospital, School of Medicine, Shanghai Jiao Tong University, Shanghai 200127, P.R. China.

**Keywords:** hsa_circ_0003823, miR-607, crisp3, apatinib, esophageal squamous cell carcinoma

## Abstract

**Background:** Circular RNAs (CircRNAs) have attracted a growing interest of research in cancer. The regulatory roles and mechanisms of circRNAs in progression, metastasis and drug resistance of esophageal squamous cell carcinoma (ESCC) needed to be clarified. Our previous study revealed the crucial role of Apatinib in ESCC therapy. However, the correlation between circRNAs and Apatinib resistance remained unclear.

**Methods:** 3 pairs of tumor and paracancerous tissues of ESCC patients were used for RNA sequencing. Western blot analysis, RNA immunoprecipitation (RIP), dual-luciferase reporter assays, apoptosis and animal assays were conducted to confirm the roles and specific mechanisms of hsa_circ_0003823 as well as the effects of it on Apatinib sensitivity in ESCC.

**Results:** Our results revealed that hsa_circ_0003823 was highly expressed in ESCC and associated with poor prognosis. Further results indicated that hsa_circ_0003823 promoted proliferation and metastasis ability of ESCC. In the section of mechanism experiments, hsa_circ_0003823 regulated CRISP3 by targeting microRNA-607 (miR-607) to promote progression of ESCC. Besides, we found that silencing hsa_circ_0003823 improved Apatinib sensitivity. hsa_circ_0003823 resulted in Apatinib resistance by miR-607/CRISP3 axis.

**Conclusions:** In this study, we elucidated the function of hsa_circ_0003823 and its role in promoting tumor progression, metastasis and Apatinib resistance of ESCC through miR-607/CRISP3 axis.

## Background

Esophageal cancer (EC) is one of the most prevalent malignant tumor worldwide, especially in China 1, 2. Esophageal squamous cell cancer is the major histopathological subtype of esophageal cancer and accounts for 90% of EC patients 3. Although significant advances have been achieved in the diagnosis and treatment strategies, the 5-year survival rates of ESCC patients with advanced stage remain poor due to early recurrence and metastasis 4, 5. For advanced ESCC patients, targeted therapy might provide a good choice for comprehensive treatment of ESCC and be an important supplement to chemotherapy 6-8. Our previous study focused on Apatinib, a novel vascular endothelial growth factor receptor-2 (VEGFR-2) tyrosine kinase inhibitor, and elucidated its crucial role in inhibiting progression and metastasis as well as sensitizing paclitaxel in ESCC. However, drug resistance was still the problem we needed to confront and tackle 9. Therefore, it has become extremely important to clarify the specific molecular mechanisms of progression, metastasis and Apatinib resistance for ESCC and discover effective therapeutic targets.

Circular RNAs (CircRNAs) are a novel and large class of non-coding RNAs with a covalently closed loop structure that are produced by backsplicing. Most circRNAs belong to non-coding RNAs (ncRNAs) without 5′ to 3′ polarity or 3′ polyA tail and are formed by a single exon or multiple exons of protein-coding genes 10. CircRNAs are mainly located in the cytoplasm, and not easily degraded by the exonuclease RNase R 11. Recently, many studies have revealed that circRNAs were involved in the occurrence, development and drug resistance in cancer 12-17. CircRNAs have many important non-coding functions, mainly including acting as miRNA sponges, interacting with miRNAs, regulating gene transcription, and interacting with RNA-binding protein to regulate other RNAs 18-20. In addition, a subset of circRNAs performs independent translation functions under certain conditions, although the vast majority of circRNAs are considered non-coding 21, 22.

MicroRNAs are short non-coding RNAs with 19-25 nucleotides that are able to inhibit translation of mRNAs and participate in multiple cellular processes, including cell cycle, proliferation, apoptosis, invasion and migration 23. It has been reported that miRNAs are involved in various cellular signaling pathways and their dysfunction could lead to occurrence and progression of cancer 24. miR-607 has been reported as a tumor suppressor. In pancreatic ductal adenocarcinoma, low serum miR-607 levels were regarded as a prognostic biomarker 25. In non-small cell lung cancer, inhibition of miR-607 promoted tumorigenesis and invasion of cancer cells 26. And in osteosarcoma, miR-607 was associated with tumor proliferation 27. However, the role of miR-607 in ESCC has not been reported.

Cysteine-rich secretory protein 3 (CRISP3) is a member of cysteine-rich secretory proteins and preferentially expressed in pancreas, prostate and salivary of human 28, 29. CRISP3 has been reported to be associated with inflammation and innate immunity 30. Recent studies revealed that CRISP3 was inextricably linked to cancer. In prostate cancer, CRISP3 could drive invasion and migration of cancer cells 31. In non-small cell lung cancer, decreasing CRISP3 expression levels inhibited progression and development of cancer 32. And in mammary carcinoma, patients with low expression levels of CRISP3 had a favorable prognosis 33. In ovarian cancer, CRISP3 was reported to be associated with drug resistance 34. So far, there were not relevant studies of CRISP3 in ESCC.

In this study, we first used RNA-seq to screen the differential expression of circRNAs between tumor and paracancerous tissues, and identified a new circular RNA—circCEP70 from CEP70 that was significantly up-regulated in ESCC, the circBase ID of which was hsa_circ_0003823. Subsequently, we discussed the clinical significance of hsa_circ_0003823, deeply illustrated the role of hsa_circ_0003823 in the occurrence and development of ESCC, and explored the underlying molecular mechanisms. The results showed that hsa_circ_0003823 was significantly up-regulated in ESCC tissues, which was related to the pathological stage and prognosis of ESCC patients, and positively related to the expression of CRISP3. Further functional and mechanistic studies have shown that hsa_circ_0003823 could act as a sponge for miR-607, alleviating its inhibition on the target gene CRISP3, thereby promoting tumor progression, metastasis and Apatinib resistance. This study explored the expression, function, regulatory mechanism, and drug resistance of hsa_circ_0003823 in ESCC for the first time, which might provide new ideas and directions for diagnosis and prognosis of ESCC.

## Methods

### Patient samples

We collected a total of 38 pairs of tumor and paracancerous tissues of ESCC patients from the Department of Thoracic Surgery, Renji Hospital affiliated to Shanghai Jiaotong University School of Medicine, of which 3 pairs were used for RNA sequencing. All tissue samples were snap-frozen in liquid nitrogen and stored at -80°C until use. All enrolled patients signed informed consent before surgery, had no history of other malignancies, and had not received chemoradiotherapy. We analyzed basic clinical data of the patients, including age, gender, TNM stage, and tumor size. This study was approved by the Ethics Committee of Shanghai Jiao Tong University School of Medicine.

### Cell culture and transfection

Human ESCC cell lines (TE-1, TE-13, ECA-109, EC9706, KYSE-150) and human esophageal epithelial cell line Het-1A as well as 293T cell line were purchased from American Type Culture Collection (ATCC) (Manassas, VA, USA). TE-1, TE-13, ECA-109, EC9706, KYSE-150 and 293T cell lines were cultured in DMEM or RPMI-1640 medium (Sigma, St. Louis, MO, USA) with 10% FBS (Gibco, USA), 100 U/ml penicillin (Millipore, TMS-AB2-C) and 100 U/ml streptomycin at 37°C with 5% CO2 in a humidified incubator. Het-1A cells were cultured in Bronchial epithelial cell basal medium (BEGM) with all the additives (Lonza, MD, USA). We used an intermittent stepwise selection protocol to establish Apatinib-resistant ESCC cells (ECA-109/AR and KYSE-150/AR cells) from ECA-109 and KYSE-150 cells over 6 months and confirmed the half maximal inhibitory concentration (IC50) according to the dose-response curves.

Circ siRNAs, CRISP3 siRNAs and miRNA mimics/inhibitors were synthesized by Bioegene (Shanghai, China) and transfected into the ESCC cell lines using lipofectamine 3000 (Invitrogen, USA). These experiments were conducted in accordance with the manufacturer's protocols. The full-length cDNA of hsa_circ_0003823 was synthesized and inserted into the expression vector pcDNA3.1 (Bioegene, Shanghai, China), while there was no hsa_circ_0003823 sequences in the mock vector which was considered as the negative control. Then cells were treated with puromycin (Sigma, USA) until hsa_circ_0003823 overexpressed cells were stably constructed. Sequences of circ siRNA, CRISP3 siRNA and miRNA mimics and inhibitors used in this study were listed in Table S1.

### RNA isolation and qRT-PCR

Total RNA was extracted from ESCC tissues and cell lines applying TRIzol reagent (Invitrogen, Carlsbad, CA, USA). Reverse transcription was carried out by the HiScript III RT SuperMix (Vazyme, Nanjing, China), and AceQ universal SYBR qPCR Master Mix (Vazyme, Nanjing, China) was used to detect total RNA under recommended conditions. GAPDH was used as the internal reference for mRNA and circRNA, and U6 was used for miRNA. Sequences of primers were listed in Table S2.

### Fluorescence *in situ* hybridization (FISH)

We performed FISH to evaluate the subcellular location of hsa_circ_0003823 using ESCC cell lines (ECA-109 and KYSE-150). After pre-hybridization was conducted at 55 °C for 2 h, cell slides were incubated with specific Cy3-labeled hsa_circ_0003823 probe (Bioegene, Shanghai, China) at 37 °C overnight and stained with DAPI. The slides were photographed with the fluorescence microscope (Leica, Germany).

### Tissue microarray (TMA) and *in situ* hybridization (ISH)

We conducted *in situ* hybridization with a specific digoxin-labeled circRNA probe to detect the relative expression of hsa_circ_0003823 (Servicebio, Wuhan, China) on TMAs (Superbiotec, Shanghai, China), containing 60 paraffin-embedded ESCC samples. TMAs were digested with proteinase K, hybridized with the specific hsa_circ_0003823 probe overnight at 4 ℃, and then treated with anti-Digoxin-AP at 4 ℃ (Roche, Basel, Switzerland). Tissues were stained with NBT/BCIP and qualified (Roche, Basel, Switzerland).

### Cell proliferation and apoptosis assays

For cell counting kit-8 (Dojindo, Japan) assays, 2x10^3 ESCC cells (ECA-109 or KYSE-150 cells) and Apatinib-resistant ESCC cells (ECA-109/AR or KYSE-150/AR cells) were plated in the 96-well plates for 5 days after cells were transfected for 48 h and the absorbance at 450 nm was measured by microplate reader. For colony formation assays, 1x10^3 ECA-109 or KYSE-150 cells were seeded in the 6-well plates and incubated for approximately 2 weeks, then stained and fixed by 1% crystal violet for 15 minutes before being photographed and counted. For apoptosis assays, ESCC cells (ECA-109 and KYSE-150) and Apatinib resistant cells (ECA-109/AR and KYSE-150/AR) were collected after transfection and stained using 3 μl FITC-Annexin V and 5 μl propidium iodide (PI, 50 μg/ml) for 15 minutes. FACS Caliber system (BD Biosciences, USA) was used to analyze apoptosis data. We set three independent events for each group.

### Cell invasion and migration assays

Transfected ESCC cells (5x10^4 cells) were suspended in serum-free medium and seeded in the top chambers. The lower chambers were added into 700 μL medium with 10% fetal bovine serum. After 24 h, ESCC cells migrated from the top chambers were fixed with 1% crystal violet for 15 minutes and then photographed and counted. We set three independent events for each group.

### Western blot analysis

Protein samples from cells or tissues which were lysed by RIPA buffer were subjected to 10% SDS-PAGE and then transferred onto polyvinylidene fluoride membranes. Primary antibodies including anti-CRISP3, anti-N-Cadherin, anti-E-Cadherin, anti-β-catenin, anti-Vimentin, anti-Snail were used (Table S3). GAPDH was used as the internal control.

### RNA immunoprecipitation (RIP)

RIP was conducted using Magna RIP kit (Millipore, MA, USA) in accordance with the manufacturer's instructions. miR-607 mimics or miR-NC were transfected into ECA-109 cells and cells were collected after 48 h and then lysed in 100 % RIP lysis buffer. RIP lysates were incubated with magnetic beads conjugated with anti-Argonaute2 (AGO2) (Millipore, MA, USA) or IgG antibody (Millipore, MA, USA) as the negative control. qRT-PCR and Western blot were used to detect the enrichment of the immunoprecipitated RNA and protein.

### Xenograft tumor model and immunochemistry

Male BALB/c nude mice (4-6 weeks) were purchased from the institute of zoology, Chinese Academy of Sciences of Shanghai and all animal experiments were performed strictly in accordance with the Guide for the Care and Use of Laboratory Animals and approved by the Committee of Animals Use and Care of Shanghai Jiaotong University School of Medicine (Approval ID: A-2018-024). ECA-109 cells were transfected using overexpression and mock vector and selected by puromycin to construct stably over-expressed cell lines. Lentiviruses (Bioegene, Shanghai, China) carrying sh-NC and sh-circ were transfected into ECA-109 cell lines, which were then selected with puromycin to obtain sh-NC and sh-circ cell lines which were named LV NC and LV circ0003823, respectively. 2 x 10^6 transfected ECA-109 cells were subcutaneously inoculated into the right flanks of male BALB/c nude mice (n=4 for each group). Mice in the Apatinib group received the drug (60mg/kg) daily by oral gavage. Tumor volume was measured once a week and calculated by the formula: V=0.5×length×width^2^. All mice were sacrificed after 4 weeks and xenograft tumors were removed to be weighed and fixed for immunohistochemistry (IHC) staining.

IHC analysis was performed according to the manufacturer's instructions (Immunostain SP kit, Dako Cytomation, USA). Primary antibodies against Ki67 (Cell Signaling Technology), β-catenin (Signalway Antibody), CRISP3 (Proteintech), E-cadherin (Cell Signaling Technology) were used. IHC results were judged by staining intensity and number of positive cells and assessed by at least three pathologists in a single-blind method.

### Dual-luciferase reporter assays

The sequences of hsa_circ_0003823, CRISP-3'UTR, and the matched mutant sequences without miR-607 binding sites were synthesized and cloned into the pmirGLO luciferase reporter vector (Promega, Madison, WI, USA). All plasmids were transfected into HEK-293T cells. Dual Luciferase Assay Kit (Promega, Madison, WI, USA) were used to measure the relative luciferase activities according to the manufacturer's protocols.

### Statistical analysis

SPSS 20.0 (IBM, SPSS, Chicago, IL, USA) and GraphPad Prism 7.0 (GraphPad Software Inc., CA, USA) were used to analyze data which were expressed as mean ± standard deviation (SD). Student's *t* test, one-way ANOVA and χ2 test were used to assess differences between groups. Kaplan-Meier method was used to evaluate survival rates. Pearson correlation was used to analyze the correlation between groups. A receiver operating characteristic (ROC) curve was used to assess the diagnostic value. *P* value < 0.05 was considered statistically significant.

## Results

### CircRNA and mRNA expression profiles in ESCC

**Figure 1:**
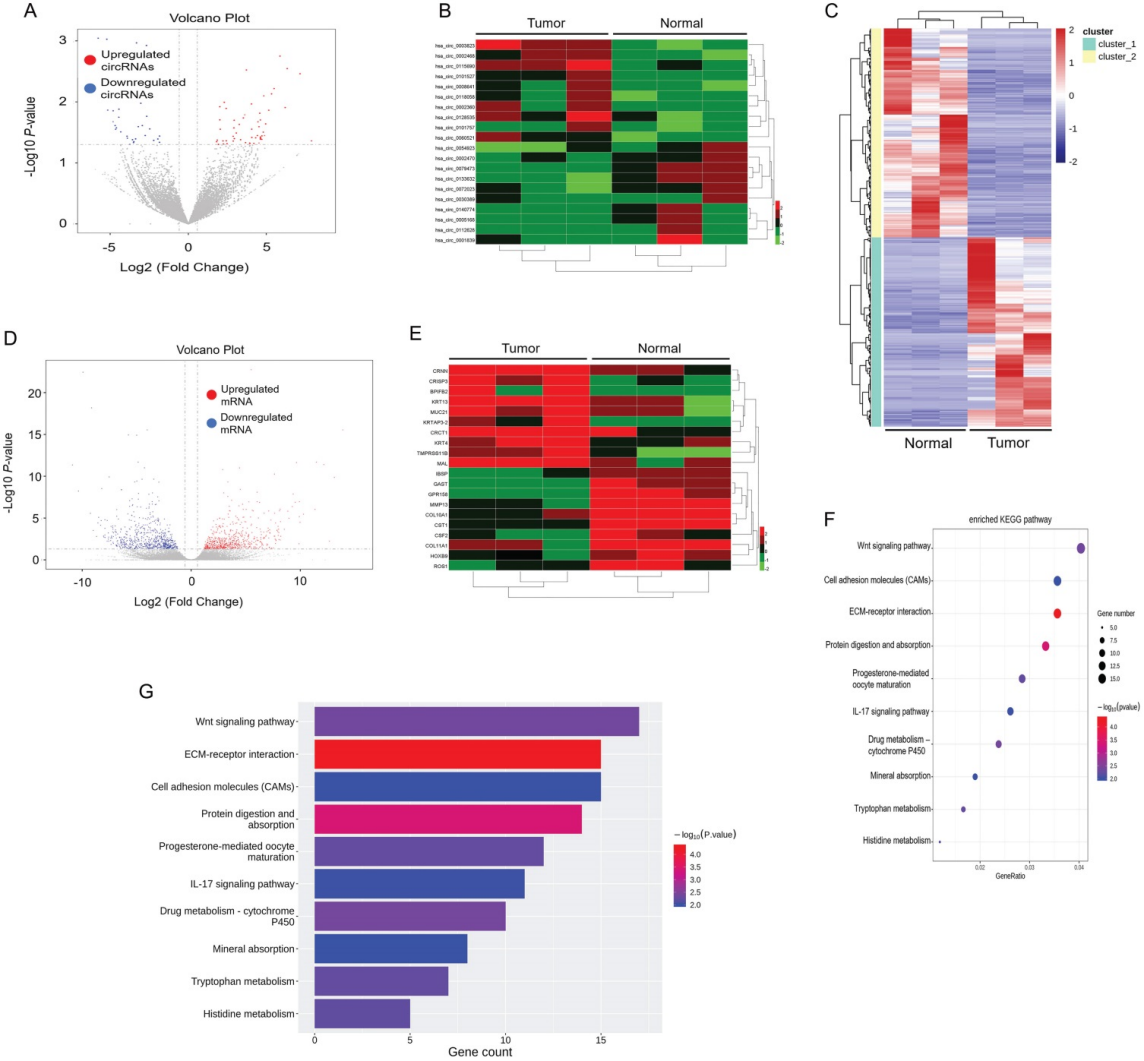
** circRNA and mRNA expression profiles in ESCC. (A)** Volcano plots showed the expression of circRNAs detected by RNA-seq in ESCC tumor and paracancerous tissues. Red and blue dots represented statistically significant up- and down-regulated circRNAs, respectively. **(B)** Heatmap showed the 10 circRNAs with the most significant up- and down-regulation in tumor group compared with paracancerous group. **(C-D)** Heatmap and volcano plots indicated the mRNA expression in ESCC tumor and paracancerous tissue by RNA-seq, with red and blue dots representing statistically significant up- and down-regulated mRNAs, respectively. **(E)** Heatmap showed the 10 most significant up- and down-regulated mRNAs in tumor group compared with paracancerous group. **(F-G)** KEGG enrichment analysis results showed the most enriched pathways.

First, we elucidated the expression of mRNA and circular RNA by RNA-sequencing of tumor tissues and paracancerous tissues in 3 ESCC patients. By setting the criteria of fold change > 2.0 and *p* value < 0.05, we found that a total of 333 circRNAs were differentially expressed in tumor and paracancerous tissues, of which 212 circRNAs were significantly up-regulated, and 121 circRNAs were down-regulated (Figure 1A). We annotated the 20 circRNAs that were most up- or down-regulated in tumor tissues in Figure 1B as a heatmap. Among them, hsa_circ_0003823 was the most up-regulated circular RNA. In addition, we also analyzed mRNA sequencing results. According to the same screening criteria, a total of 1424 mRNAs were found and differentially expressed in tumor tissues and paracancerous tissues, of which 677 mRNAs were up-regulated in tumor tissues, while 747 mRNAs were down-regulated (Figure 1C and 1D). 20 mRNAs with the most pronounced up- or down-regulation were shown in a heatmap format (Figure 1E). Through Kyoto Encyclopedia of Genes and Genomes (KEGG) enrichment analysis of sequencing results, Wnt signaling pathway and cell molecules adhesion (CAMs) were the most enriched signaling pathways (Figure 1F and 1G), and CRISP3 was the most significantly up-regulated gene in tumor tissues and closely related to tumor progression and metastasis. Tumor metastasis played an important role in the occurrence and development of ESCC. Most deaths of ESCC patients were due to tumor cells spreading to other organs, proliferating and resisting conventional treatment, eventually leading to failure of important organs 35. Therefore, this study mainly focused on the roles and mechanisms of hsa_circ_0003823 and CRISP3 in ESCC progression and metastasis.

### hsa_circ_0003823 and CRISP3 were highly expressed in ESCC and both were associated with poor prognosis

**Figure 2:**
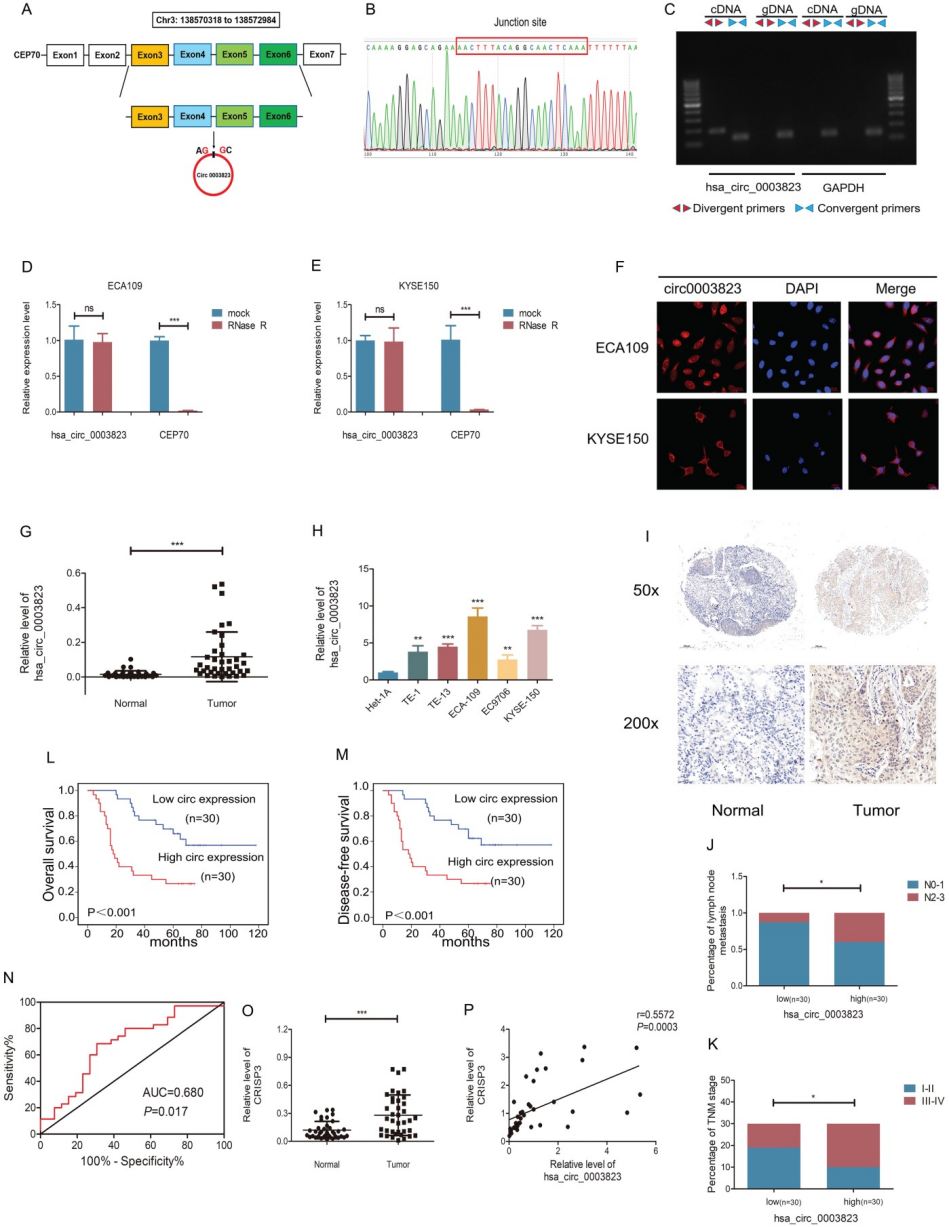
** hsa_circ_0003823 and CRISP3 were highly expressed in ESCC and both were associated with poor prognosis. (A)** Schematic diagram showed the circling process of hsa_circ_0003823 in exons 3-6 of chromosome 3 of CEP70. **(B)** Sanger sequencing on the PCR products of hsa_circ_0003823. **(C)** hsa_circ_0003823 and GAPDH were amplified with divergent and convergent primers by using the cDNA and gDNA templates in 293T cells. **(D-E)** qRT-PCR was used to detect the levels of hsa_circ_0003823 in ECA-109 (D) and KYSE-150 (E) cells with or without RNase R. **(F)** The subcellular localization of hsa_circ_0003823 in ECA-109 and KYSE-150 cells was evaluated by FISH experiments. **(G-H)** qRT-PCR was performed to detect relative levels of hsa_circ_0003823 in tumor and paracancerous tissues (n=38) of ESCC (G) or cell lines (H). **(I)** ISH assays were used to detect hsa_circ_0003823 expression levels in tumor and paracancerous tissues of ESCC and representative images were shown. **(J)** Percentages of specimen of low (n=30) and high (n=30) hsa_circ_0003823 group according to N stage. **(K)** Percentages of specimen of low (n=30) and high (n=30) hsa_circ_0003823 group according to TNM stage. **(L-M)** Kaplan-Meier survival curves of overall survival (L) and disease-free survival (M) were used to assess effects of hsa_circ_0003823 levels (high expression group: n=30; low expression group: n=30) on survival time in 60 ESCC patients. **(N)** ROC curve was used to evaluate diagnostic value of hsa_circ_0003823. **(O)** qRT-PCR was used to detect relative levels of CRISP3 in tumor and paracancerous tissues of ESCC (n=38). **(P)** Pearson correlation analysis of CRISP3 and hsa_circ_0003823 in ESCC tissues was performed. * p<0.05, ** p<0.01, *** p<0.001.

hsa_circ_0003823 consisted of 522 nucleotides and was derived from the splicing of exons 3, 4, 5, and 6 of the CEP70 gene, located at Chr3:138570318 to 138572984, also known as circCEP70 (Figure 2A). To evaluate the existence of hsa_circ_0003823, we first amplified it in 293T cells, and then performed Sanger sequencing on the PCR products, which confirmed that the head-to-tail splicing of the PCR products was in conformity to the expected size and site (Figure 2B). Considering that head-to-tail splicing was not only the result of reverse splicing of cDNA, but also the result of gene rearrangement, we designed polymer primers and divergent primers for hsa_circ_0003823 respectively, and amplified them with cDNA or gDNA of 293T cells. Results showed that hsa_circ_0003823 could only be amplified from cDNA, not gDNA (Figure 2C). In addition, the stability was also an important feature of circular RNAs 11, 36, 37. To confirm the stability of hsa_circ_0003823, RNase R was used to treat ECA-109 and KYSE-150 cell lines, and the results indicated that the expression level of CEP70 in the RNase R group was significantly reduced, while hsa_circ_0003823 expression was not obviously affected (Figure 2D and 2E). Since the function of circular RNA was usually related to its localization in cells, we further conducted FISH experiments to explore intracellular localization of hsa_circ_0003823 in ESCC cell lines, and the results showed that hsa_circ_0003823 was mostly localized in the cytoplasm (Figure 2F). Next, qRT-PCR was used to detect the expression level of hsa_circ_0003823 in 38 pairs of ESCC tumor and paracancerous tissues, and we found that the expression level of hsa_circ_0003823 was significantly higher in tumor tissues (Figure 2G). Then we divided 38 pairs of ESCC patients into high-expression group and low-expression group according to levels of hsa_circ_0003823, and further analyzed their clinical characteristics. The results showed that patients in high-expression group had higher T stage, N stage and TNM stage. However, there was no difference in age, gender and tumor size (Table 1). Besides, we also detected the expression level of hsa_circ_0003823 in ESCC cell lines (TE-1, TE-13, ECA-109, EC9706, KYSE-150) and the human esophageal epithelial cell line Het-1A, and the results indicated that the expression level was higher in the ESCC cell lines, especially ECA-109 and KYSE-150, so we chose the above two cell lines for subsequent experiments (Figure 2H). To further verify our conclusions, we performed *in situ* hybridization on tissue chips containing 60 ESCC tumor tissues and paracancerous tissues to detect the expression of hsa_circ_0003823, and the results indicated that the expression level was significantly higher in tumor tissues (Figure 2I). We divided 60 ESCC samples into high hsa_circ_0003823 expression group (n=30) and low expression group (n=30). We found that the high expression group had a higher proportion of lymph node metastasis and III-IV of TNM stage (Figure 2J and 2K). The Kaplan-Meier survival curve showed that the high expression group had significantly shorter overall survival and progression-free survival time than low expression group (Figure 2L and 2M). The ROC curve revealed that hsa_circ_0003823 had high sensitivity in distinguishing tumor tissues from paracancerous tissues (Figure 2N). In addition, we also examined the expression level of CRISP3 in 38 pairs of ESCC tumor and paracancerous tissues, and the results showed that the expression level of CRISP3 was significantly higher in tumor tissues (Figure 2O). Pearson correlation analysis revealed a positive correlation between the expression of hsa_circ_0003823 and CRISP3 (Figure 2P). The above results confirmed the RNA-seq data and pointed out that hsa_circ_0003823 and CRISP3 might play a synergistic role in ESCC progression.

### hsa_circ_0003823 promoted proliferation, migration and invasion of ESCC cells

**Figure 3:**
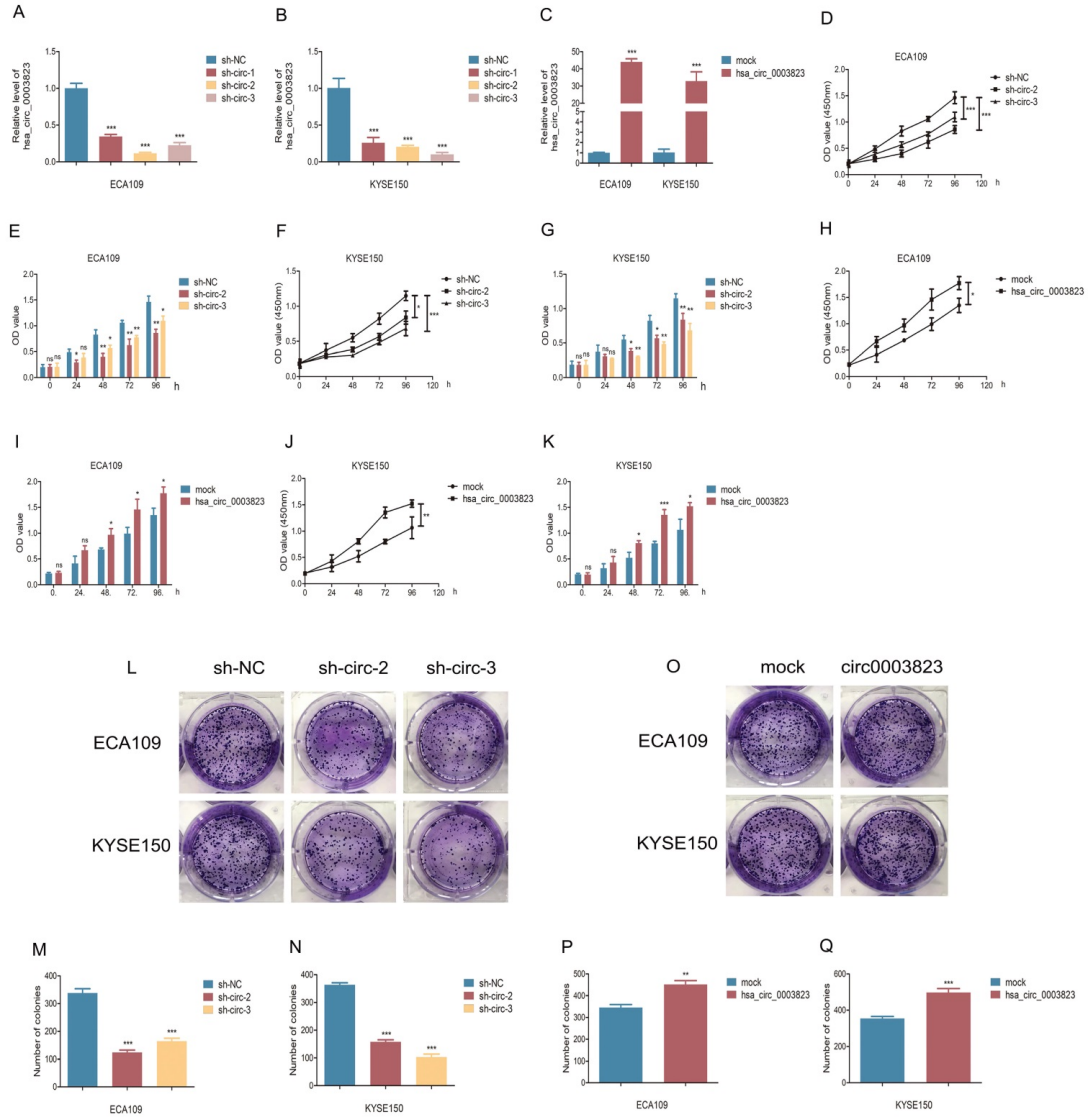

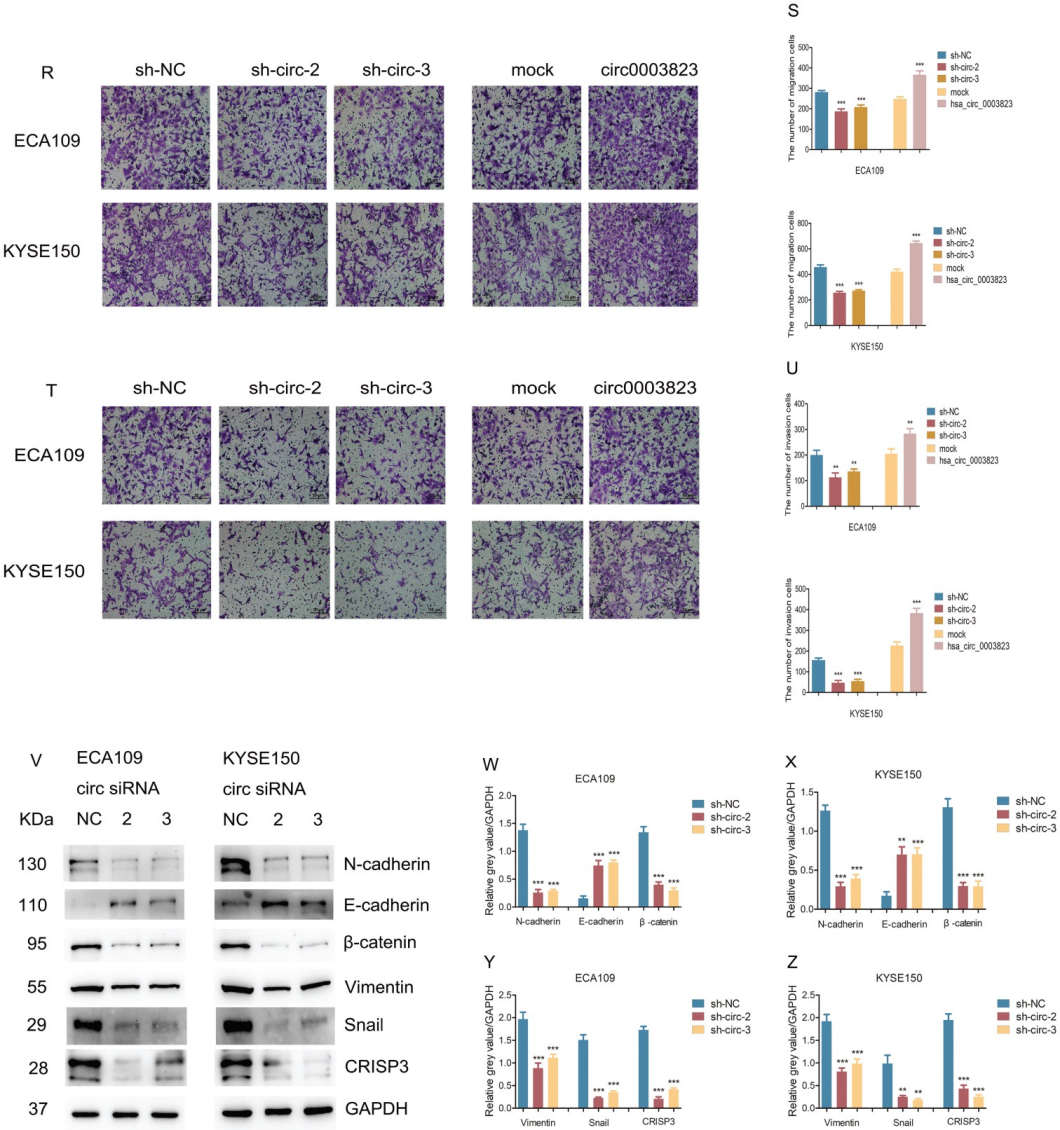
** hsa_circ_0003823 promoted proliferation, migration and invasion of ESCC cells. (A-C)** Construction of hsa_circ_0003823 knockdown (A-B) and overexpression (C) cell lines. qRT-PCR was used to detect the levels of hsa_circ_0003823 in ECA-109 and KYSE-150 cells. **(D-K)** Cell viability was evaluated by CCK8 in ECA-109 and KYSE-150 cells with hsa_circ_0003823 knockdown (D-G) or overexpression (H-K). **(L-Q)** Clone formation experiments were carried out in ECA-109 and KYSE-150 cells with hsa_circ_0003823 knockdown (L-N) or overexpression (O-Q). **(R-S)** Migration assays were performed in ECA-109 and KYSE-150 cells with hsa_circ_0003823 knockdown or overexpression. Results were analyzed. **(T-U)** Invasion assays were performed in ECA-109 and KYSE-150 cells with hsa_circ_0003823 knockdown or overexpression. Results were analyzed. **(V-Z)** The expression levels of metastasis-related genes were detected by Western blot. Results were analyzed. * p<0.05, ** p<0.01, *** p<0.001.

To explore the role of hsa_circ_0003823 in ESCC cells, we first constructed hsa_circ_0003823 knockdown and overexpression cell lines, and qRT-PCR was used to validate the knockdown and overexpression effects of hsa_circ_0003823 in ECA-109 and KYSE-150 cell lines (Figure 3A-3C). For the knockdown experiments, we designed 3 shRNA sequences named sh-circ-1, sh-circ-2 and sh-circ-3. qRT-PCR results showed that the knockdown effects of sh-circ-2 and sh-circ-3 were better than sh-circ-1, so we chose the former for subsequent experiments. Next, we evaluated the effects of hsa_circ_0003823 on the proliferation of ESCC cell lines using CCK8 assays. The results showed that the up-regulation of hsa_circ_0003823 significantly enhanced the proliferation of cells, while its down-regulation inhibited the proliferation of cells (Figure 3D-3K). Plate colony formation experiments were used to further validate our conclusions, showing that knockdown of hsa_circ_0003823 reduced the number of plate clones, whereas overexpression of it significantly increased the number of plate clones (Figure 3L-3Q). Figure 3R-3U indicated knockdown of hsa_circ_0003823 significantly reduced the ability of ESCC cells for invasion and migration, while overexpression of hsa_circ_0003823 increased the number of cells undergoing invasion and migration. The effects of knockdown of hsa_circ_0003823 on the expression levels of tumor metastasis-related proteins were investigated by Western blot experiments.

The results revealed that knockdown of hsa_circ_0003823 increased the expression level of E-cadherin and inhibited expression levels of N-cadherin, β-catenin, Vimentin and Snail (Figure 3V-3Z). The above results exhibited that hsa_circ_0003823 could enhance the proliferation, invasion and migration ability of ESCC cells. In addition, we also found that the expression level of CRISP3 was reduced after knockdown of hsa_circ_0003823, which further confirmed the correlation between the two.

### hsa_circ_0003823 facilitated tumorigenesis and metastasis of ESCC *in vivo*

**Figure 4:**
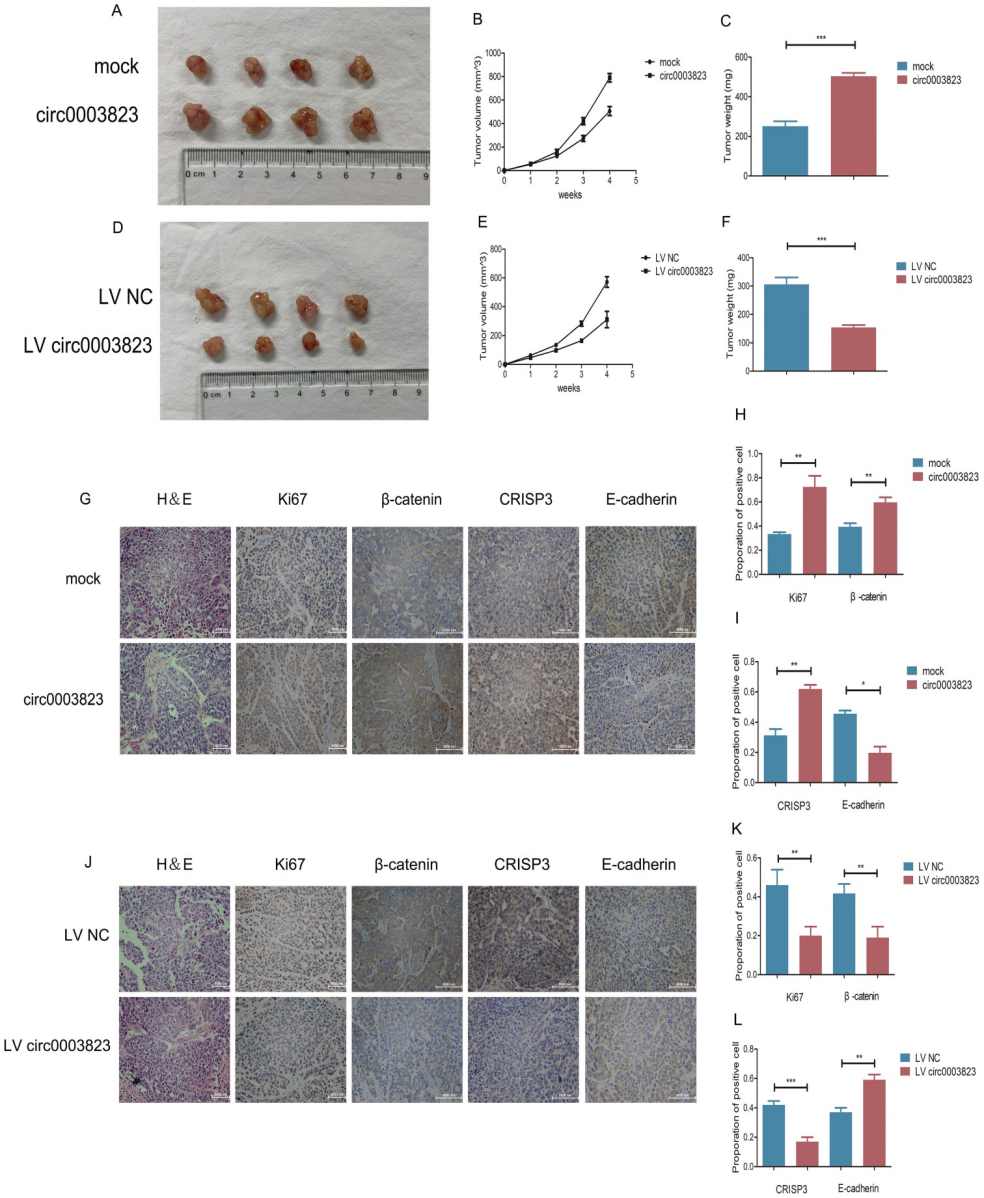
** hsa_circ_0003823 facilitated tumorigenesis and metastasis of ESCC *in vivo*. (A)** Representative pictures of xenograft tumors in control and circ0003823 overexpression group were shown. **(B)** Tumor volume in control and circ0003823 overexpression group was assessed each week for four weeks. **(C)** Tumor weight in control and circ0003823 overexpression group was evaluated. **(D)** Representative pictures of xenograft tumors in control and circ0003823 knockdown group were shown. **(E)** Tumor volume in control and circ0003823 knockdown group was assessed each week for four weeks. **(F)** Tumor weight in control and circ0003823 knockdown group was evaluated. **(G-L)** IHC staining of Ki67, β-catenin, CRISP3 and E-cadherin were conducted and results were analyzed. * p<0.05, ** p<0.01, *** p<0.001.

To verify the role of hsa_circ_0003823 in proliferation, invasion and migration of ESCC *in vivo*, we subcutaneously injected ECA-109 cells with knockdown or overexpression of hsa_circ_0003823 into adult male nude mice. The results showed that, compared with the control group, the subcutaneous tumorigenicity in hsa_circ_0003823 overexpression group was higher (Figure 4A). The volume and weight of tumors in the overexpression group were also higher than those in the control group (Figure 4B and 4C). However, the subcutaneous tumorigenicity, tumor volume and weight were all lower in hsa_circ_0003823 knockdown group than that of the control group (Figure 4D-4F). IHC results indicated that the expression levels of Ki67 and β-catenin in the tumor tissues of the hsa_circ_0003823 overexpression group were significantly higher than the control group, while the E-cadherin level was lower in the hsa_circ_0003823 overexpression group (Figure 4G-4I). The expression levels of Ki67, β-catenin and E-cadherin in the hsa_circ_0003823 knockdown group were contrary to the above results (Figure 4J-4L). In addition, we also detected the expression level of CRISP3 and found that overexpression of hsa_circ_0003823 increased the expression level of CRISP3, while knockdown of hsa_circ_0003823 resulted in downregulation of CRISP3 (Figure 4G-4L). The above results revealed the importance of hsa_circ_0003823 in the occurrence and development of ESCC *in vivo*, which might be related to CRISP3 and metastasis-related signaling pathways.

### hsa_circ_0003823 acted as the sponge for miR-607 to inhibit it and activated the expression of CRISP3

**Figure 5:**
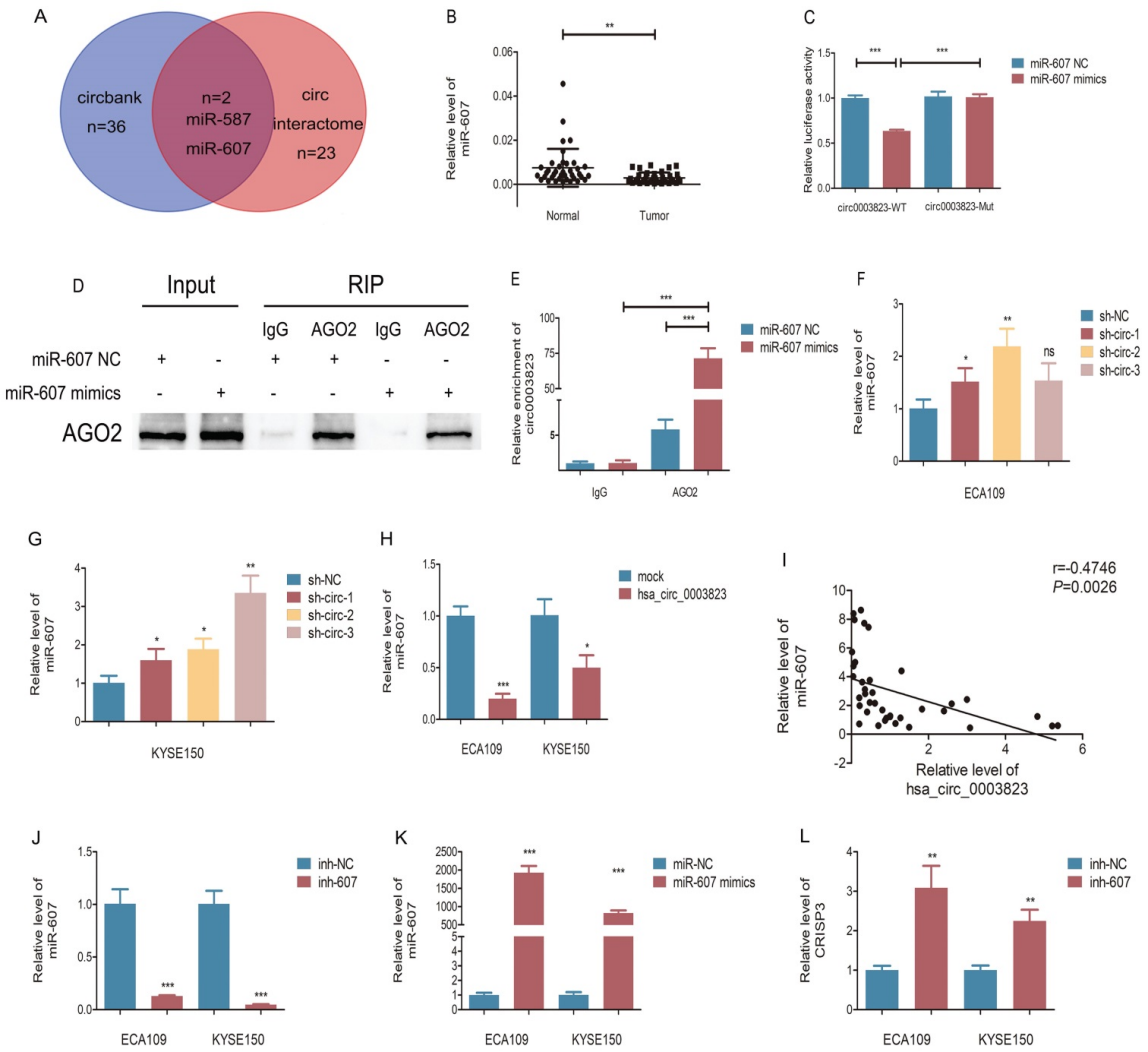

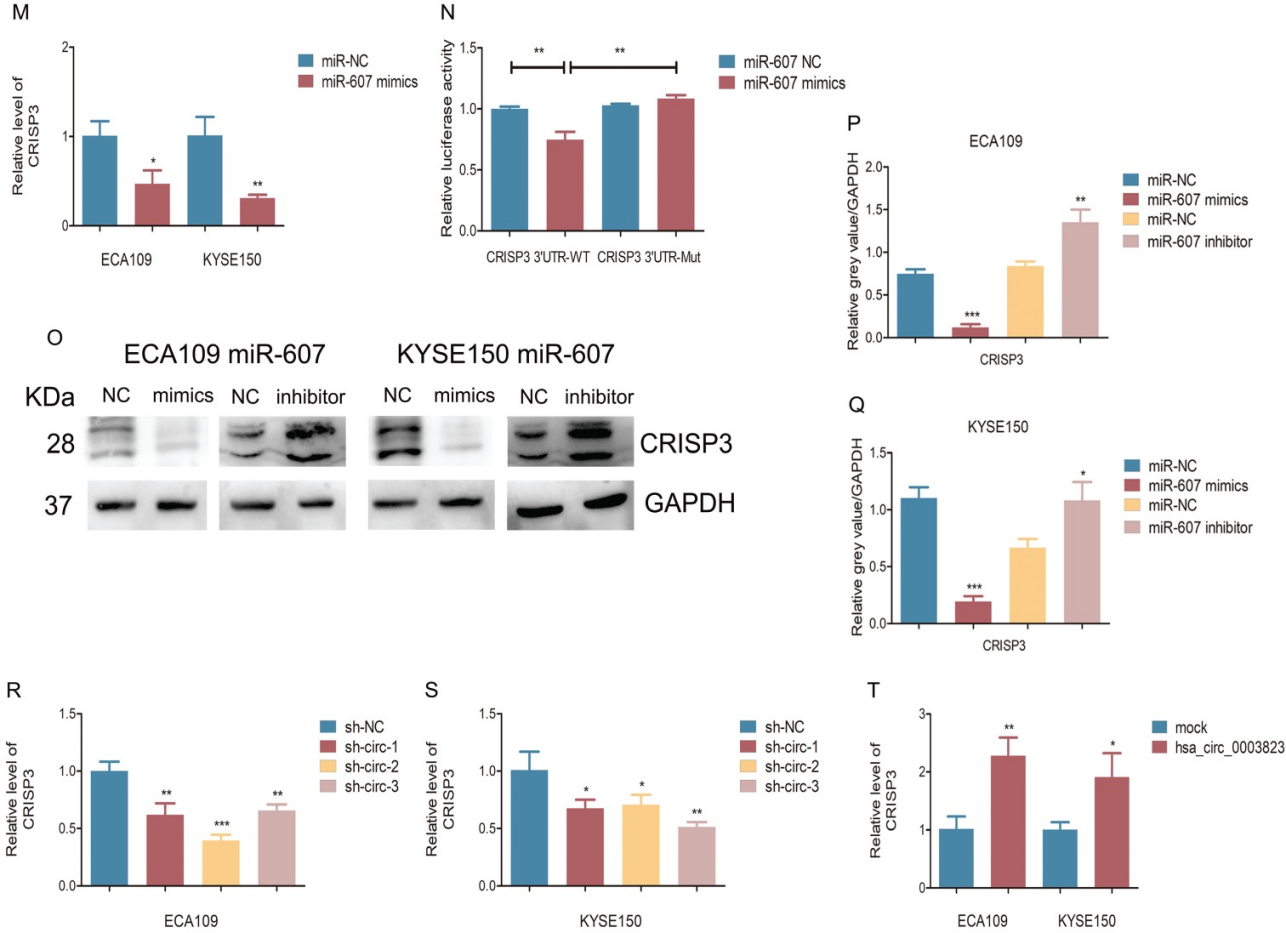
** hsa_circ_0003823 acted as the sponge for miR-607 to inhibit it and activated the expression of CRISP3. (A)** Schematic diagram showed intersection of miRNAs predicted by different databases that could bind to hsa_circ_0003823. **(B)** qRT-PCR was used to detect relative levels of miR-607 in tumor and paracancerous tissues of ESCC (n=38). **(C)** Relative luciferase activities were evaluated after 293T cells were transfected with hsa_circ_0003823-WT or hsa_circ_0003823-Mut and miR-607 NC or miR-607 mimics. **(D-E)** Anti-AGO2 RNA immunoprecipitation (RIP) assays were performed after ECA-109 cells were transfected with miR-607 NC or miR-607 mimics. AGO2 protein and miR-607 were detected by Western blot and qRT-PCR, respectively. **(F-H)** Relative levels of miR-607 were measured by qRT-PCR after ECA-109 and KYSE-150 cells were transfected with indicated vectors. **(I)** Pearson correlation analysis of miR-607 and hsa_circ_0003823 in ESCC tissues was performed. **(J-M)** Relative levels of miR-607 and CRISP3 were detected by qRT-PCR after ECA-109 and KYSE-150 cells were transfected with inh-NC and inh-607, miR-NC and miR-607 mimics. **(N)** Relative luciferase activities were evaluated after 293T cells were transfected with CRISP3 3'UTR-WT or CRISP3 3'UTR-Mut and miR-607 NC or miR-607 mimics. **(O-Q)** Relative expression levels of CRISP3 were detected by Western blot after ECA-109 and KYSE-150 cells were transfected with miR-NC and miR-607 inhibitor, miR-NC and miR-607 mimics. **(R-T)** Relative levels of CRISP3 were detected by qRT-PCR after ECA-109 and KYSE-150 cells were transfected with indicated vectors. * p<0.05, ** p<0.01, *** p<0.001.

In order to elucidate the downstream molecular mechanism of hsa_circ_0003823 in ESCC, we first predicted the potential miRNA targets of hsa_circ_0003823 through circbank and circ interactome databases. The results showed that hsa_circ_0003823 had a targeted binding site on the sequences of miR-607 (Figure 5A). Next, we used qRT-PCR to detect the expression level of miR-607 in 38 pairs of ESCC tumor and paracancerous tissues, and found that the expression level of miR-607 in tumor tissues was lower (Figure 5B). To verify the targeting effect of hsa_circ_0003823 on miR-607, we performed a dual-luciferase reporter experiment in 293T cells. The full-length sequences of hsa_circ_0003823 of wild type or mutant type without miR-607 binding sites were subcloned into the luciferase reporter vector pmirGLO, which was transfected into ESCC cells, and then transfected with miR-607 NC and mimics, respectively. The results showed that compared with the control group, miR-607 mimics could significantly reduce the luciferase activity in the wild type group, while there was no significant effect on the mutant group, indicating that there was a direct interaction between hsa_circ_0003823 and miR-607 (Figure 5C). In addition, we verified our conclusions by anti-AGO2 RNA immunoprecipitation (RIP) assays in ECA-109 cells. The results showed that compared with the IgG group, AGO2 antibody could obviously pull down hsa_circ_0003823. And compared with the miR-607 NC group, cells transfected with miR-607 mimics could enrich hsa_circ_0003823 more efficiently (Figure 5D and 5E). hsa_circ_0003823 was knocked down in ECA-109 and KYSE-150 cell lines and the expression level of miR-607 was detected by qRT-PCR. We found that compared with the control group, the expression levels of miR-607 in the hsa_circ_0003823 knockdown groups increased (Figure 5F and 5G). Conversely, overexpression of hsa_circ_0003823 in ESCC cell lines suppressed the expression level of miR-607 (Figure 5H). Pearson correlation analysis revealed a negative correlation between hsa_circ_0003823 and miR-607 expression in 38 pairs of ESCC tumor tissues (Figure 5I).

Our previous results have shown that hsa_circ_0003823 was positively correlated with the expression of CRISP3, and hsa_circ_0003823 could act as a miR-607 sponge to inhibit its function. To explore the interaction among hsa_circ_0003823, miR-607 and CRISP3, we used the TargetScan database for prediction and found that miR-607 was the target miRNA of hsa_circ_0003823, and CRISP3 was the target gene of miR-607 (http://www.targetscan.org/vert_72/). miR-607 inhibitor was used to inhibit its expression (Figure 5J), and miR-607 mimics for increasing its expression (Figure 5K). qRT-PCR results showed that compared with the control group, miR-607 inhibitor significantly increased the level of CRISP3, while miR-607 mimics reduced the level of CRISP3 (Figure 5L and 5M). The dual luciferase reporter assay showed that compared with the CRISP3 3'UTR-Mut group, miR-607 mimics could obviously reduce the fluorescence intensity of the luciferase reporter vector carrying the CRISP3 3'UTR-WT sequences (Figure 5N). Western blot results showed that compared with the control group, miR-607 inhibitor could significantly increase the expression level of CRISP3, while miR-607 mimics could inhibit the expression level of CRISP3 (Figure 5O-5Q). qRT-PCR was used to detect the level of CRISP3 in the previously constructed hsa_circ_0003823 knockdown or overexpression ESCC cell lines. Results indicated that compared with the control group, knockdown of hsa_circ_0003823 inhibited the level of CRISP3, while overexpression of hsa_circ_0003823 increased the level of CRISP3 (Figure 5R-5T).

The above results suggested that hsa_circ_0003823 acted as a miR-607 sponge to inhibit it and activate the expression of CRISP3 to promote tumor progression of ESCC.

### hsa_circ_0003823 promoted invasion and migration of ESCC through miR-607/CRISP3 axis

**Figure 6:**
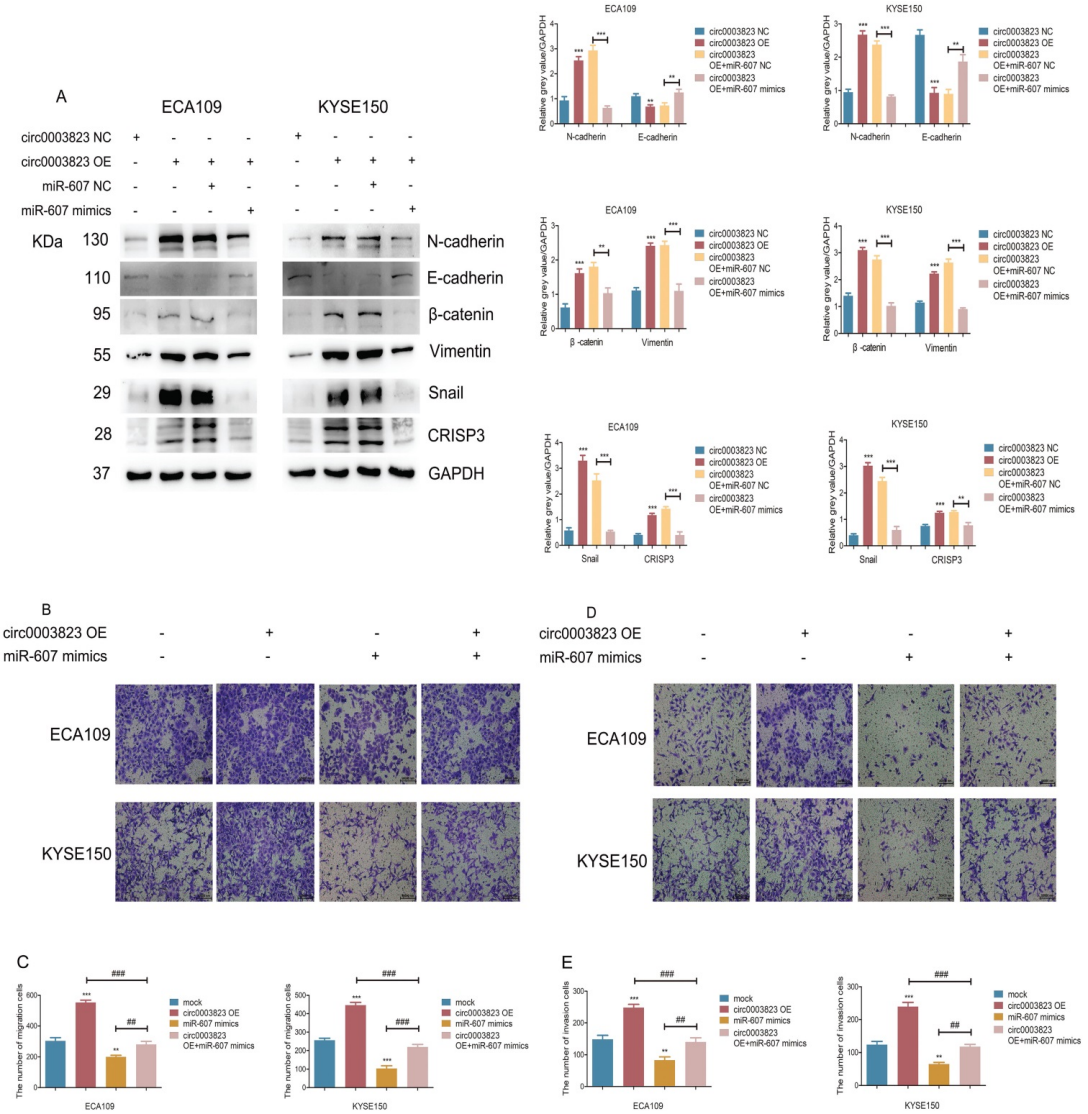

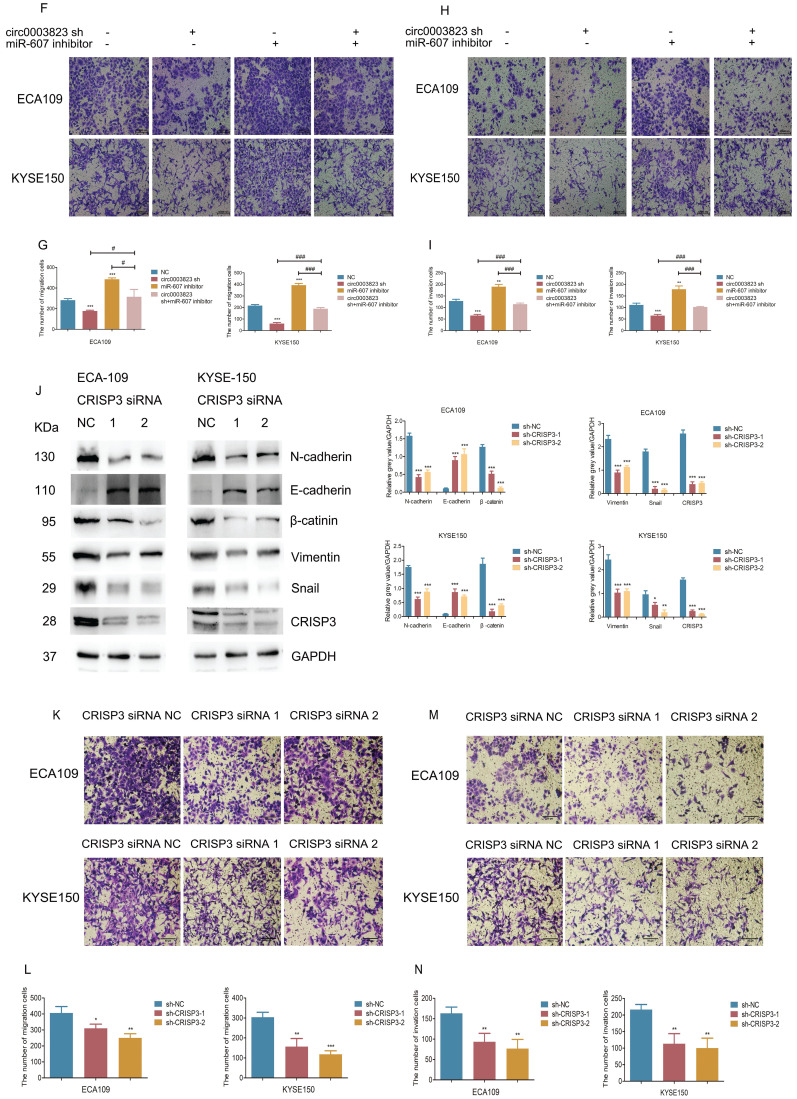

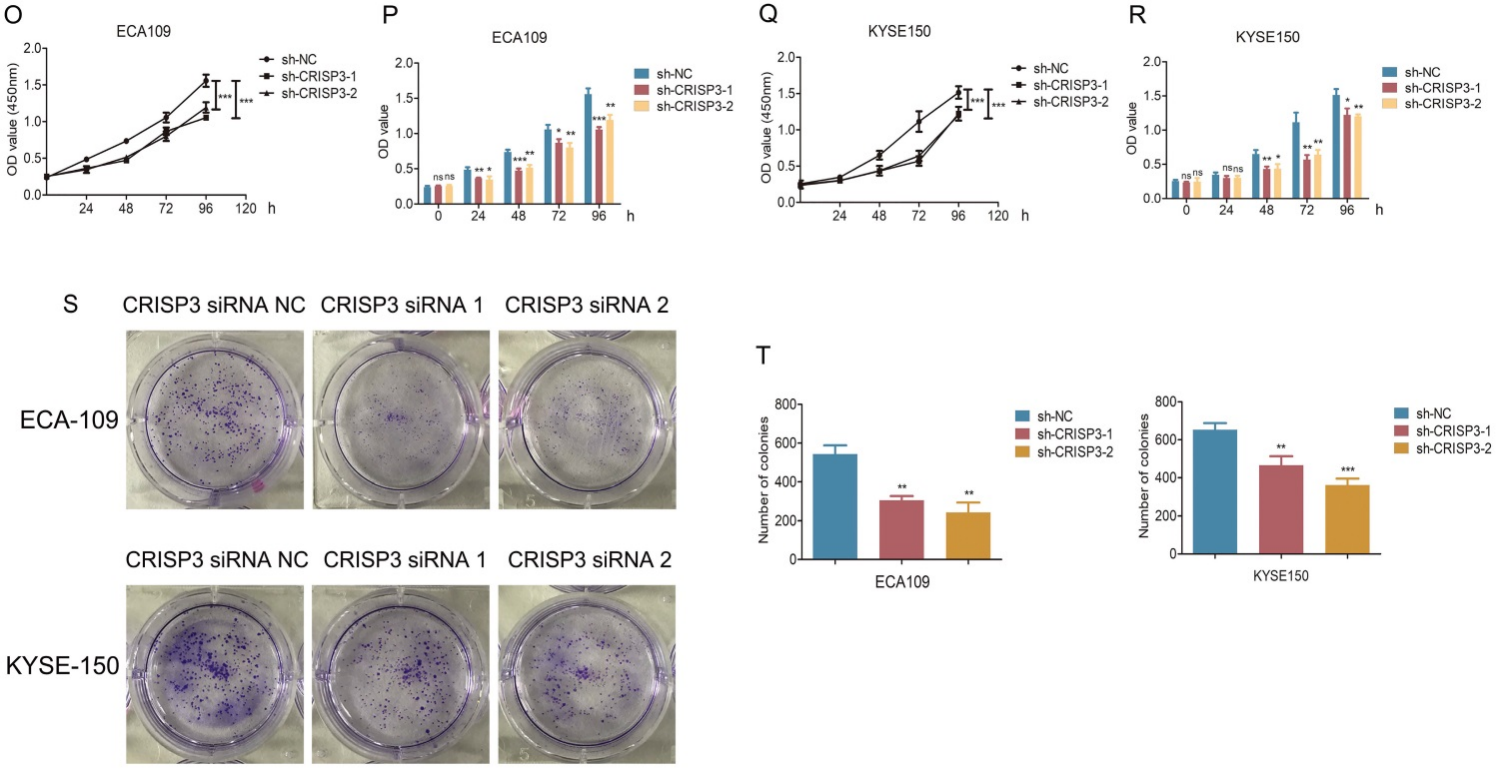
** hsa_circ_0003823 promoted invasion and migration of ESCC through miR-607/CRISP3 axis. (A)** Western blot was used to detect expression levels of metastasis-related genes and CRISP3 after ECA-109 and KYSE-150 cells were transfected with mock or hsa_circ_0003823 OE, miR-607 NC or miR-607 mimics. **(B-C)** Migration assays were performed after ECA-109 and KYSE-150 cells were transfected with mock or hsa_circ_0003823 OE, miR-607 NC or miR-607 mimics. Results were analyzed. **(D-E)** Invasion assays were performed after ECA-109 and KYSE-150 cells were transfected with mock or hsa_circ_0003823 OE, miR-607 NC or miR-607 mimics. Results were analyzed. **(F-G)** Migration assays were performed after ECA-109 and KYSE-150 cells were transfected with NC or hsa_circ_0003823 sh, miR-607 NC or miR-607 inhibitor. Results were analyzed. **(H-I)** Invasion assays were performed after ECA-109 and KYSE-150 cells were transfected with NC or hsa_circ_0003823 sh, miR-607 NC or miR-607 inhibitor. Results were analyzed. **(J)** Western blot was used to detect expression levels of metastasis-related genes and CRISP3 after ECA-109 and KYSE-150 cells were transfected with NC or CRISP3 siRNA. **(K-L)** Migration assays were performed after ECA-109 and KYSE-150 cells were transfected with NC or CRISP3 siRNA. Results were analyzed. **(M-N)** Invasion assays were performed after ECA-109 and KYSE-150 cells were transfected with NC or CRISP3 siRNA. Results were analyzed. **(O-R)** Cell viability was evaluated by CCK8 for CRISP3-knockdown ECA-109 and KYSE-150 cells. **(S-T)** Clone formation experiments were carried out for CRISP3-knockdown ECA-109 and KYSE-150 cells. * p<0.05, ** p<0.01, *** p<0.001.

In order to explore the specific mechanism of hsa_circ_0003823 exerting its biological function, miR-607 mimics were transfected into ESCC cell lines under the premise of overexpression of hsa_circ_0003823. Western blot results showed that overexpression of hsa_circ_0003823 up-regulated the expression levels of N-cadherin, β-catenin, Vimentin, Snail, and down-regulated the expression level of E-cadherin, while the transfection of miR-607 mimics reversed the above phenomena (Figure 6A). In addition, we found that the upregulation of CRISP3 caused by overexpression of hsa_circ_0003823 was also reversed by the transfection of miR-607 mimics (Figure 6A). The results of migration and invasion experiments showed that overexpression of hsa_circ_0003823 could promote cell migration and invasion, while the miR-607 mimics group not only inhibited the migration and invasion ability of ESCC cells, but also reversed the increase in the number of migrating and invading cells due to hsa_circ_0003823 (Figure 6B-6E). Moreover, the results of cell migration and invasion experiments indicated that knockdown of hsa_circ_0003823 could reduce the migration and invasion ability of cells, while inhibiting the expression of miR-607 could increase the number of migrating and invading cells, and reverse the reduction in the number of migrating and invading cells caused by knockdown of hsa_circ_0003823 (Figure 6F-6I).

CRISP3 siRNAs were transfected into ESCC cell lines to reveal the function of CRISP3 in this process. Western blot results indicated that knockdown of CRISP3 down-regulated the expression levels of N-cadherin, β-catenin, Vimentin, Snail, and up-regulated the expression level of E-cadherin (Figure 6J). The results of cell migration and invasion experiments showed that knockdown of CRISP3 reduced the migration and invasion ability of cells (Figure 6K-6N). CCK8 assays were performed to evaluated the effects of CRISP3 on the proliferation of ESCC cell lines. The results showed that knockdown of CRISP3 significantly inhibited the proliferation of cells (Figure 6O-6R). Plate colony formation experiments were conducted to further validate our conclusions, indicating that knockdown of CRISP3 reduced the number of plate clones (Figure 6S-6T).

The above results suggested that hsa_circ_0003823 promoted progression, invasion and migration of ESCC through miR-607/CRISP3 axis.

### hsa_circ_0003823 regulated the sensitivity of ESCC cells to Apatinib through miR-607/CRISP3 axis *in vitro* and *in vivo*

**Figure 7:**
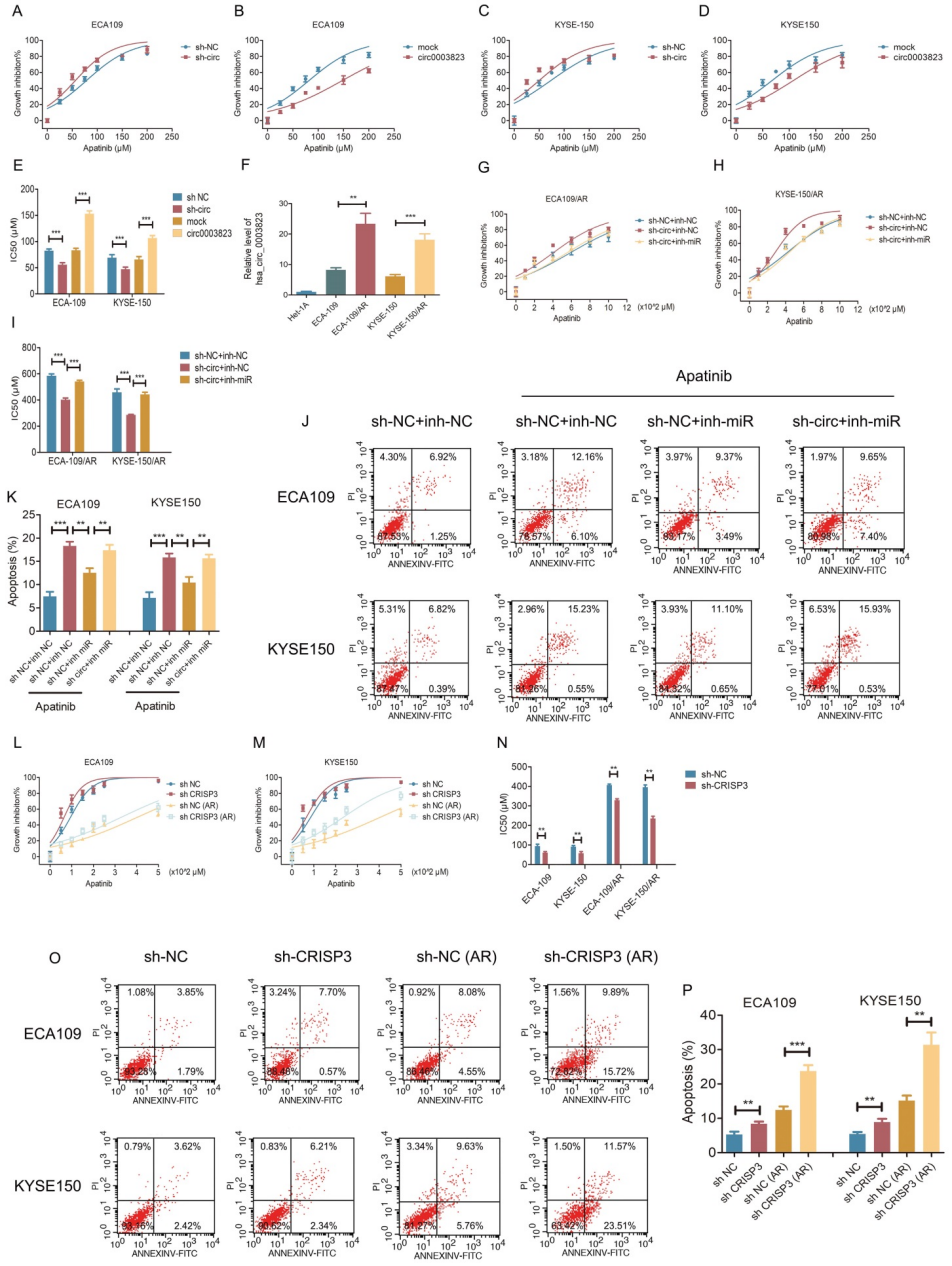
** hsa_circ_0003823 regulated the sensitivity of ESCC cells to Apatinib through miR-607/CRISP3 axis *in vitro*. (A-E)** Cell viability was evaluated by CCK8 for hsa_circ_0003823 knockdown or overexpression ESCC cells treated with Apatinib. **(F)** Relative levels of CRISP3 were detected by qRT-PCR in different groups. **(G-I)** Cell viability was evaluated by CCK8 for miR-607 inhibiting or hsa_circ_0003823 knockdown ESCC cells treated with Apatinib. **(J-K)** Apoptosis experiments were evaluated by Annexin V-FITC and propidium iodide (PI) staining for miR-607 inhibiting or has_circ_0003823 knockdown ESCC cells treated with Apatinib. **(L-N)** Cell viability was evaluated by CCK8 for CRISP3-knockdown normal or Apatinib-resistant ESCC cells treated with Apatinib. **(O-P)** Apoptosis experiments were evaluated by Annexin V-FITC and propidium iodide (PI) staining for CRISP3-knockdown normal or Apatinib-resistant ESCC cells treated with Apatinib. * p<0.05, ** p<0.01, *** p<0.001.

To test the effects of hsa_circ_0003823 on Apatinib sensitivity, we set 7 different Apatinib concentrations to treat previously constructed hsa_circ_0003823 knockdown and overexpression cell lines (ECA-109 and KYSE-150). The results showed that inhibiting hsa_circ_0003823 significantly weakened the viability of Apatinib-treated cells, while overexpression of hsa_circ_0003823 obviously enhanced Apatinib resistance (Figure 7A-7D). IC50 values were calculated and presented in Figure 7E. To further explore the regulation of hsa_circ_0003823 on Apatinib sensitivity, we constructed Apatinib-resistant ECA-109/AR and KYSE-150/AR cells. Compared with normal ESCC cell lines, the expression levels of hsa_circ_0003823 were significantly increased in Apatinib-resistant cells (Figure 7F). ECA-109/AR and KYSE-150/AR cells were transfected with hsa_circ_0003823 siRNA or miR-607 inhibitor and then treated with different concentrations of Apatinib. Results indicated that knockdown of hsa_circ_0003823 weakened viability of Apatinib-treated ESCC/AR cells, while inhibiting miR-607 was able to reverse the above phenomenon (Figure 7G-7I). Flow cytometry with double staining of Annexin V and PI was used to analyze the effects of hsa_circ_0003823 knockdown or miR-607 inhibitor on the apoptosis of ECA-109 and KYSE-150 cell lines treated with Apatinib, and the results showed that inhibiting miR-607 decreased the apoptosis ratio of ESCC cell lines, while knockdown of hsa_circ_0003823 could reverse the effects of miR-607 inhibitor (Figure 7J-7K). CRISP3 siRNA was transfected into normal and Apatinib-resistant ESCC cells. Results indicated that knockdown of CRISP3 decreased viability of both normal and Apatinib-resistant ESCC cells and increased apoptosis rates (Figure 7L-7P).

**Figure 8:**
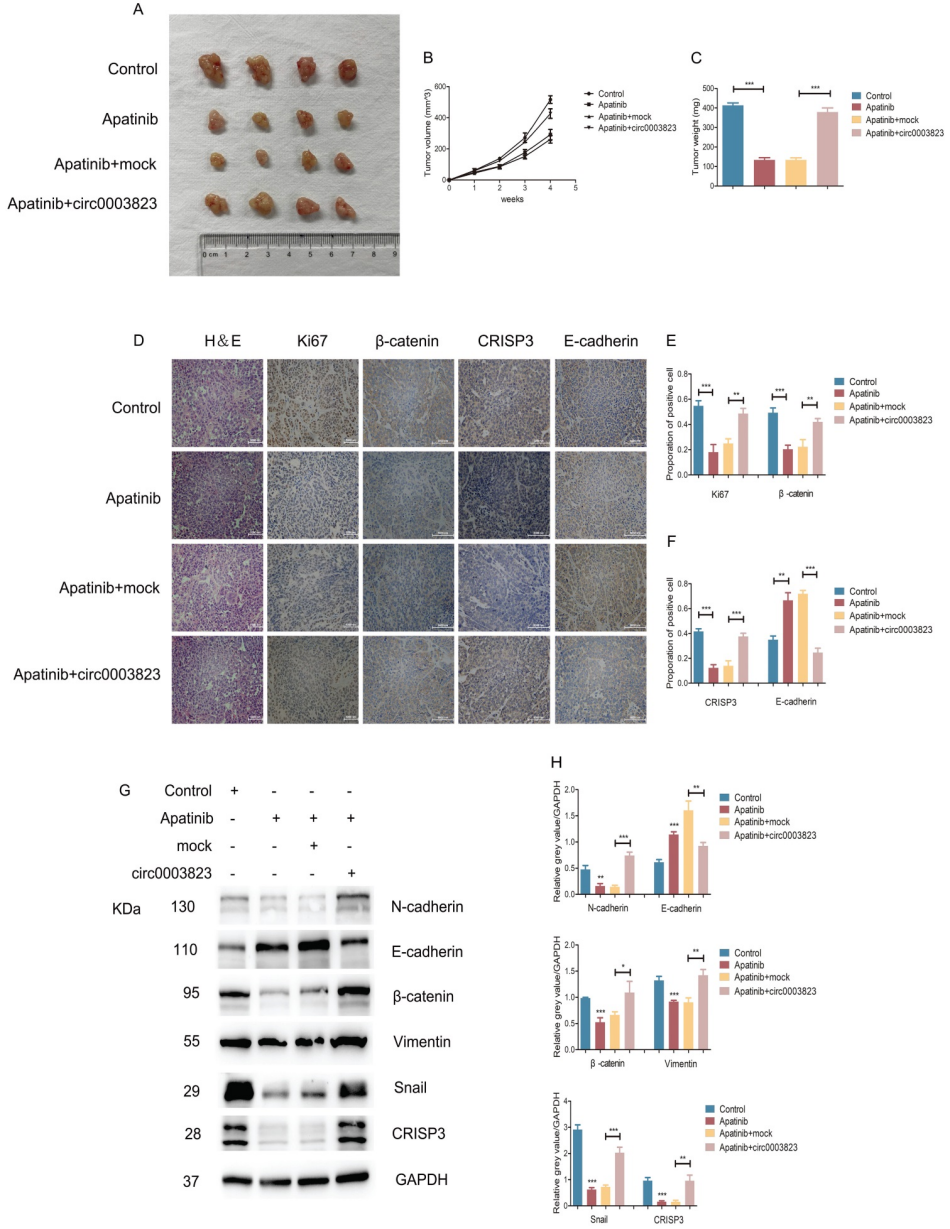

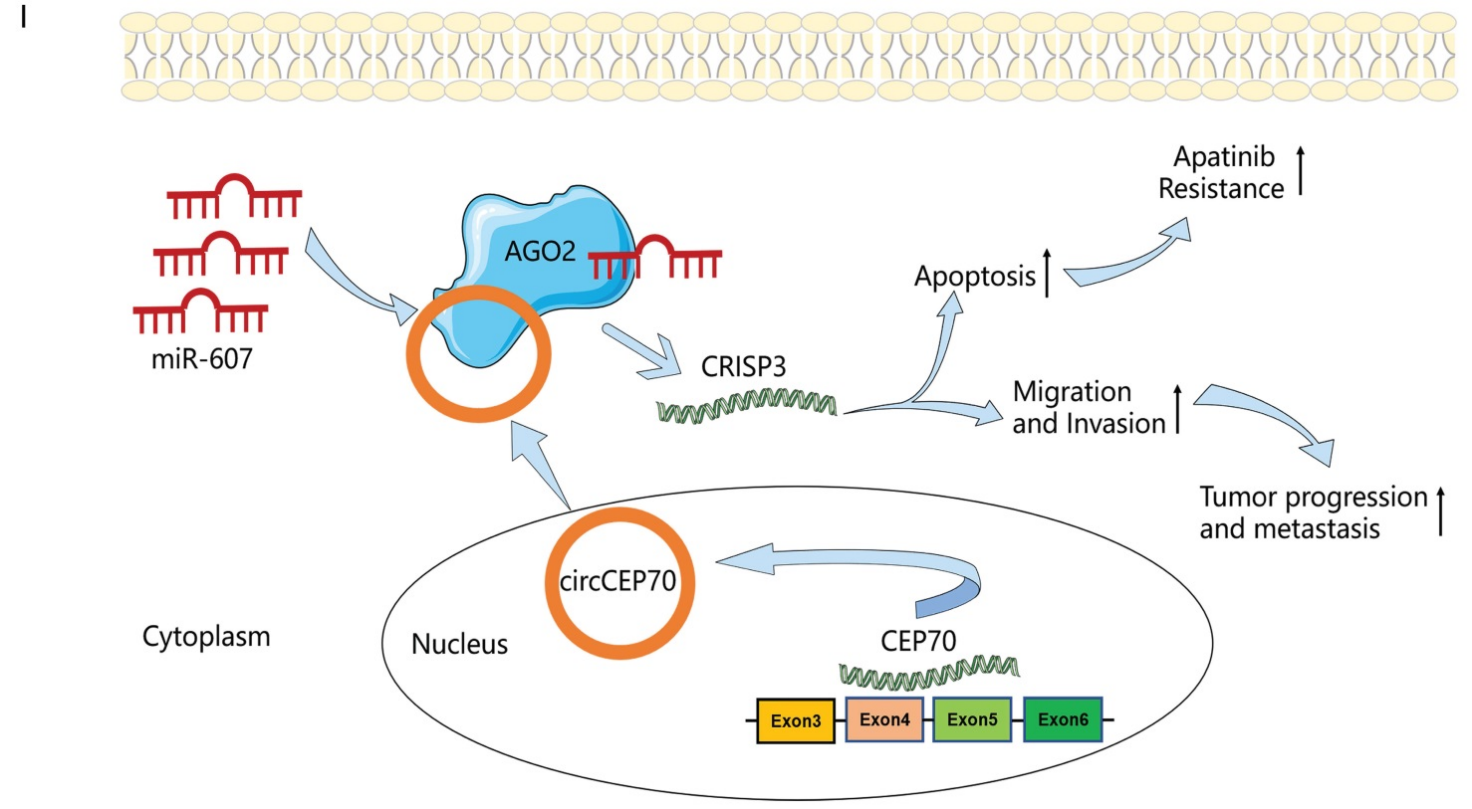
** hsa_circ_0003823 regulated the sensitivity of ESCC cells to Apatinib *in vivo*. (A)** Representative pictures of xenograft tumors in control, Apatinib, Apatinib+mock and Apatinib+circ0003823 group were shown. **(B)** Tumor volume in control, Apatinib, Apatinib+mock and Apatinib+circ0003823 group was assessed each week for four weeks. **(C)** Tumor weight in control, Apatinib, Apatinib+mock and Apatinib+circ0003823 group was evaluated. **(D-F)** IHC staining of Ki67, β-catenin, CRISP3 and E-cadherin were conducted and results were analyzed. **(G-H)** The metastasis-related genes were detected by Western blot. Results were analyzed. **(I)** Schematic diagram. * p<0.05, ** p<0.01, *** p<0.001.

We also validated the above conclusions by *in vivo* experiments. The normal ECA-109 cell line or cells transfected with mock or hsa_circ_0003823 was subcutaneously injected into adult male nude mice. After two weeks, all mice were assigned into 4 groups: control group, Apatinib group, Apatinib and mock group, Apatinib and circ0003823 group. All the mice were sacrificed four weeks after the drugs administration, and the tumors were removed to measure the volume and weight. The results showed that the tumorigenicity of ECA-109 cells in Apatinib group was significantly reduced, and the tumor volume and weight were significantly lower than those of the control group (Figure 8A-8C). Overexpression of hsa_circ_0003823 could reverse the inhibitory effects of Apatinib on tumor (Figure 8A-8C). IHC results showed that the expression levels of Ki67, β-catenin and CRISP3 in the Apatinib group were significantly decreased, while the expression level of E-cadherin was increased. Similarly, the above phenomenon was reversed by overexpression of hsa_circ_0003823 (Figure 8D-8F). Western blot was performed to further verify our conclusions and results showed that Apatinib could inhibit expression levels of N-cadherin, β-catenin, Vimentin, Snail and CRISP3, increase the level of E-cadherin, while overexpression of hsa_circ_0003823 reversed the above phenomenon (Figure 8G-8H).

The above *in vitro* and *in vivo* experiments confirmed that hsa_circ_0003823 regulated the sensitivity of ESCC cells to Apatinib through miR-607/CRISP3 axis *in vitro* and *in vivo*.

## Discussion

ESCC is one of the most malignant tumor with high incidence and lethality, which is prone to early distant metastasis and drug resistance, and the regulatory mechanism of ESCC is still unclear 3, 38-40. In recent years, there have been a large amount of studies on circular RNAs, and now we have better understanding of the roles of circular RNAs in biogenesis and biology. However, the regulatory functions and corresponding mechanisms in many diseases, especially tumors, are still not thoroughly studied 41-46. At present, there are relatively few reports on the development, metastasis and drug resistance of circRNAs in ESCC, and the specific mechanism needs to be clarified. Wang J revealed that knockdown of circ_0087378 could repress the tumorigenesis and progression of ESCC by modulating the miR-140-3p/E2F3 axis 47.

Liu Z focused on the role of circDOPEY2 and concluded that circDOPEY2 inhibited CPEB4-mediated Mcl-1 translation process and enhanced chemosensitivity of ESCC 48. Liang Y reported that CircIMMP2L promoted ESCC progression via CtBP1 nuclear retention dependent epigenetic modification 49. We discovered a novel circRNA named hsa_circ_0003823 by identifying the circRNA and mRNA expression profiles of 3 pairs of ESCC tumor and paracancerous tissues, which was also the most up-regulated circRNA in tumor tissues. Next, we detected the expression level of hsa_circ_0003823 in 38 pairs of ESCC tumor and paracancerous tissues, and determined that it was highly expressed in tumor tissues, and was closely related to the TNM stage, especially N stage of ESCC patients, Kaplan-Meier survival curve and other prognostic indicators. Further functional experiments showed that knockdown of hsa_circ_0003823 could inhibit the proliferation of ESCC cells, and weaken the ability of cells to invade and migrate, while overexpression of hsa_circ_0003823 had the opposite effects. And we also found that hsa_circ_0003823 affected expression levels of metastasis-related proteins. These experimental results indicated that hsa_circ_0003823 played an important role in the occurrence, development and metastasis of ESCC.

The downstream mechanism of circRNAs is related to the localization. CircRNAs are generally acting as ceRNAs by sponging miRNAs when localized in the cytoplasm 50-52. It was reported that circLPAR3 could act as a miR-198 sponge to promote the invasion and migration of ESCC 53. CircRNA_2646 performed the function as the ceRNA to promote progression of esophageal squamous cells by inhibiting miR-124/PLP2 signaling pathway 54. Another study found that ciRS-7 accelerated ESCC progression through acting as a miR-876-5p sponge to enhance MAGE-A family expression 55. We confirmed that hsa_circ_0003823 was mainly located in the cytoplasm of ESCC cells using FISH experiments. By predicting the potential miRNA targets of hsa_circ_0003823 using relevant databases, we found that there were targeted binding sites between hsa_circ_0003823 and miR-607. It has been reported that miR-607 plays an important role as the tumor suppressor in various tumors including pancreatic cancer, non-small cell lung cancer, and osteosarcoma 25-27, however, its role in ESCC has not yet been elucidated. We found that the expression level of miR-607 in ESCC tumor tissues was significantly lower than that in paracancerous tissues. Dual luciferase reporter and anti-AGO2 RNA immunoprecipitation (RIP) assays confirmed the interaction between hsa_circ_0003823 and miR-607. Knockdown of hsa_circ_0003823 in ESCC cell lines upregulated miR-607, while overexpression of hsa_circ_0003823 suppressed miR-607 expression. Pearson correlation analysis showed that there was a negative correlation between the two. Therefore, we speculated that hsa_circ_0003823 might act as a role in accelerating tumor progression in ESCC through sponge of miR-607.

CRISP3 is a member of the cysteine-rich secretory proteins, and it has been reported that CRISP3 was involved in the occurrence, development and drug resistance in a variety of tumors including prostate cancer, non-small cell lung cancer, breast cancer and so on 31-34, however, its role in ESCC remained unknown. Through RNA-seq in 3 pairs of ESCC tumor and paracancerous tissues and qRT-PCR experiments on 38 pairs of tumor and paracancerous tissues, we found that the expression level of CRISP3 in tumor tissues was significantly higher, and it was closely related to tumor development and metastasis. Pearson correlation analysis showed that there was a strong positive correlation between CRISP3 and hsa_circ_0003823. Knockdown or overexpression of hsa_circ_0003823 in ESCC cell lines significantly decreased or increased the expression level of CRISP3, further confirming the close relationship between the two. hsa_circ_0003823 could adsorb miR-607 through sponges and inhibit its function, thereby promoting ESCC progression. TargetScan database prediction showed that CRISP3 was one of the potential target genes of miR-607. Dual-luciferase reporter assays confirmed that miR-607 could directly target the 3-untranslated regions of CRISP3. Inhibition of miR-607 significantly increased the mRNA and protein levels of CRISP3, whereas overexpression of miR-607 had the opposite effects. Therefore, we confirmed that CRISP3 could be positively regulated by hsa_circ_0003823, which acted as the sponge of miR-607.

KEGG enrichment analysis of RNA-seq results showed that metastasis-related signaling pathways were the most enriched pathways. Therefore, in terms of mechanism exploration, we focused on whether hsa_circ_0003823 affected ESCC progression and metastasis through the miR-607/CRISP3 signaling axis. Our study showed that overexpression of hsa_circ_0003823 significantly enhanced the invasion and migration ability of tumor cells, and increased the expression levels of CRISP3 and metastasis-related proteins, while knockdown of hsa_circ_0003823 had the opposite effects. At the same time, we found that miR-607 partially reversed the changes in invasion and migration ability of ESCC and the expression levels of CRISP3 and metastasis-related proteins caused by hsa_circ_0003823. Functional experiments showed that knockdown of CRISP3 inhibited the proliferation, migration and invasion of ESCC cells, and affected expression levels of metastasis-related proteins. This study demonstrated that hsa_circ_0003823, as a ceRNA, promoted CRISP3-mediated tumor progression and metastasis in ESCC by inhibiting miR-607.

Apatinib is a novel VEGFR-2 tyrosine kinase inhibitor and has been reported not only to inhibit tumor progression but also to increase the sensitivity of tumor cells to chemotherapy drugs. Our previous study revealed the important role of Apatinib in ESCC and found that Apatinib could inhibit proliferation, migration and invasion, induce ER stress, autophagy and apoptosis, and potentiate cell sensitivity to paclitaxel in ESCC 56. However, drug resistance was prone to occurrence for advanced ESCC patients. Several studies have reported the correlation between circRNA and drug resistance. Circ0008399 was reported to promote cisplatin resistance through interacting with WTAP in bladder cancer 15. CircRNA-SORE could mediate sorafenib resistance in hepatocellular carcinoma by stabilizing YBX1 16. Another study focused on the function of circSNX6 and revealed that it could promote sunitinib resistance in renal cell carcinoma through miR-1184/GPCPD1/lysophosphatidic acid axis 14. However, the role of circRNA in drug resistance of ESCC has been rarely reported. Our study explored the effects of hsa_circ_0003823 on Apatinib sensitivity and found that inhibiting hsa_circ_0003823 significantly weakened the viability of Apatinib-treated cells through miR-607/CRISP3 axis. For *in vivo* experiments, overexpression of hsa_circ_0003823 could reverse the inhibitory effects of Apatinib on tumorigenicity. Our study confirmed that hsa_circ_0003823 regulated the sensitivity of ESCC cells to Apatinib through miR-607/CRISP3 axis *in vitro* and *in vivo*.

Of course, this study had some limitations. The entire study was based on commercially purchased ESCC cell lines, which couldn't provide the most reliable *in vivo* and *in vitro* experimental results. Therefore, if necessary, further validation of experimental results using ESCC cells derived from tumor tissues of patients or PDX models was required.

## Conclusions

In summary, in current studies, for the first time, we elucidated the function of hsa_circ_0003823 in ESCC and the underlying mechanism by regulating the expression of CRISP3 through sponge adsorption of miR-607, thereby promoting the progression, metastasis and Apatinib resistance of ESCC. Our findings suggested that hsa_circ_0003823 might be a potential biomarker and a novel target in the diagnosis and treatment of ESCC. The study of hsa_circ_0003823/miR-607/CRISP3 axis would expand our knowledge in understanding the underlying pathogenesis of ESCC.

## Supplementary Material

Supplementary tables.

## Figures and Tables

**Table 1:** Correlation between has_circ_0003823 expression and clinicopathological features in 38 ESCC patients.

Characteristic	Cases	hsa_circ_0003823		Chi-square	*P* value
		low	high		
All cases	38	19	19		
Age (years)					
>65	23	12	11	0.110	0.740
≤65	15	7	8		
Gender					
Male	28	13	15	0.546	0.460
Female	10	6	4		
T stage					
T1-T2	21	14	7	5.348	0.021
T3-T4	17	5	12		
N stage					
N0-N1	26	16	10	4.537	0.033
N2-N3	12	3	9		
TNM stage					
I-II	23	15	7	7.163	0.007
III-IV	15	4	12		
Tumor size (cm)					
>3.5	11	6	5	0.128	0.720
≤3.5	27	13	14		

## Abbreviations

circRNAs: circular RNAs
ESCC: esophageal squamous cell carcinoma
RIP: RNA immunoprecipitation
EC: esophageal cancer
RNA seq: RNA sequencing
ncRNAs: non-coding RNAs
CRISP3: Cysteine-rich secretory protein 3
VEGFR-2: Vascular endothelial growth factor receptor-2
ATCC: American Type Culture Collection
BEGM: Bronchial epithelial cell basal medium
IC50: Half maximal inhibitory concentration
FISH: Fluorescence *in situ* hybridization
TMA: Tissue microarray
ISH: *in situ* hybridization
RIP: RNA immunoprecipitation
AGO2: anti-Argonaute2
IHC: immunohistochemistry
SD: Standard deviation
ROC: receiver operating characteristic curve
CAMs: Cell molecules adhesion
circ_0003823: Hsa_circ_0003823
miR-607: MicroRNA-607
DMEM: Dulbecco's modified Eagle's medium
FBS: Fetal bovine serum
qRT-PCR: Quantitative real-time polymerase chain reaction
cDNA: Complementary DNA
gDNA: Genomic DNA
GAPDH: Glyceraldehyde-3-phosphate dehydrogenase
NC: Negative control
PBS: Phosphate-buffered saline
H&E: Hematoxylin and eosin
WT: Wild-type
MUT: Mutant
KEGG: Kyoto Encyclopedia of Genes and Genomes
