# Supplementary material for: Circular RNA hsa_circ_0003823 promotes the Tumor Progression, Metastasis and Apatinib Resistance of Esophageal Squamous Cell Carcinoma by miR-607/CRISP3 Axis
Source: Int J Biol Sci. 2022 Sep 21;18(15):5787–808. doi: 10.7150/ijbs.76096 (PMC9576509; doi:10.7150/ijbs.76096)
Supplement: Supplementary file 1 — Supplementary tables. [file ijbsv18p5787s1.pdf]

Table S1 Sequences of circ siRNA, CRISP3 siRNA and miRNA mimics and inhibitors used in this study

hsa_circ_0003823 si-1 sense	CAGAAAACUUUACAGGCAACUTT
hsa_circ_0003823 si-1 antisense	AGUUGCCUGUAAAGUUUUCUGTT
hsa_circ_0003823 si-2 sense	UACAGGCAACUCAAAUUUUUUTT
hsa_circ_0003823 si-2 antisense	AAAAAAUUUGAGUUGCCUGUATT
hsa_circ_0003823 si-3 sense	ACUUUACAGGCAACUCAAAUUTT
hsa_circ_0003823 si-3 antisense	AAUUUGAGUUGCCUGUAAAGUTT
hsa_circ_0003823 NC sense	UUCUCCGAACGUGUCACGUdTdT
hsa_circ_0003823 NC antisense	ACGUGACACGUUCGGAGAAdTdT
CRISP3 si-1 sense	AGUAACCCAAAGGAUCGAATT
CRISP3 si-1 antisense	UUCGAUCCUUUGGGUUACUTT
CRISP3 si-2 sense	GUUGGAUGUGGAAAUGCCUTT
CRISP3 si-2 antisense	AGGCAUUUCCACAUCCAACTT
CRISP3 NC sense	UUCUCCGAACGUGUCACGUdTdT
CRISP3 NC antisense	ACGUGACACGUUCGGAGAAdTdT
hsa-miR-607 mimics sense	GUUCAAAUCCAGAUCUAUAAC
hsa-miR-607 mimics antisense	GUUAUAGAUCUGGAUUUGAAC
hsa-miR NC sense	UUCUCCGAACGUGUCACGUdTdT
has-miR NC antisense	ACGUGACACGUUCGGAGAAdTdT
hsa-miR-607 inhibitor	GUUAUAGAUCUGGAUUUGAAC
has-miR NC	UUCUCCGAACGUGUCACGUdTdT

Table S2 Sequences of primers used for qPCR in this study

hsa_circ_0003823-F	AAGTAGGGCTTGCCACCAAC
hsa_circ_0003823-R	GGTTGACTGGAATCCTGGGG
hsa-miR-607 RT	CTCAACTGGTGTCGTGGAGTCG GCAATTCAGTTGAGGTTATAG
hsa-miR-607 F	ACACTCCAGCTGGGGTTCAAATCCAGATC
hsa-miR-607 R	TGGTGTCGTGGAGTCG
CRISP3-F	ACCTCGTTGGATGTGGAAAT
CRISP3-R	CAACCATTGGTGCATAGTCC
CEP70-F	GTCAACAGAACATGATACAGGAG
CEP70-R	TAGTGATTCATCCTCCAATTCAC
U6-F	CTCGCTTCGGCAGCACATATACT
U6-R	ACGCTTCACGAATTTGCGTGTC
GAPDH-F	GAAGGTGAAGGTCGGAGTC
GAPDH-R	GAAGATGGTGATGGGATTTC

Table S3 Antibodies used for western blotting in this study

Antibody	Company	Cat. No.	Source	Dilution
CRISP3	Proteintech	14847-1-AP	Rabbit	1:1000
N-Cadherin	Cell signaling	13116	Rabbit	1:1000
E-Cadherin	Cell signaling	3195	Rabbit	1:1000
β-catenin	Signalway Antibody	48621-1	Rabbit	1:1000
Vimentin	Cell signaling	5741	Rabbit	1:1000
Snail	Cell signaling	3879	Rabbit	1:1000
GAPDH	Cell signaling	5174	Rabbit	1:1000
